# Etiology of acute diarrhea in the elderly in China: A six-year observational study

**DOI:** 10.1371/journal.pone.0173881

**Published:** 2017-03-21

**Authors:** Zike Zhang, Shengjie Lai, Jianxing Yu, Qibin Geng, Wanqi Yang, Yu Chen, Jianguo Wu, Huaiqi Jing, Weizhong Yang, Zhongjie Li

**Affiliations:** 1 Department of Laboratory Medicine, First Affiliated Hospital, College of Medicine, Zhejiang University, Hangzhou, China; 2 Key Laboratory of Clinical In Vitro Diagnostic Techniques of Zhejiang Province, Hangzhou, China; 3 Division of Infectious Disease, Key Laboratory of Surveillance and Early-warning on Infectious Disease, Chinese Center for Disease Control and Prevention, Beijing, China; 4 Worldpop, Department of Geography and Environment, University of Southampton, Southampton, United Kingdom; 5 Institute of Pathogen Biology, Chinese Academy of Medical Sciences &Peking Union Medical College, Beijing, China; 6 State Key Laboratory of Virology, College of Life Sciences, Wuhan University, Wuhan, China; 7 National Institute for Communicable Diseases Control and Prevention, Chinese Center for Disease Control and Prevention, Beijing, China; Kliniken der Stadt Köln gGmbH, GERMANY

## Abstract

Acute diarrhea leads to a substantial disease burden among the elderly worldwide. However, in the context of increasingly aging trend in China, the prevalence of etiological agents among elderly diarrheal patients was undetermined. This study aimed to explore the major enteropathogens of acute diarrhea among outpatients older than 65 years in China, and also the epidemiological features of the pathogens. Demographic and clinical data for acute diarrhea among outpatients older than 65 years were collected from 213 participating hospitals from 2009 to 2014. Stool specimens were collected and tested for 13 enteric viruses and bacteria. The proportion of outpatients positive for targeted pathogens was analyzed by residential areas and seasonal patterns. Among the 7,725 patients enrolled, 1,617 (20.9%)were positive for any one of the 13 study pathogens. The predominant pathogen was norovirus (9.0%), followed by *diarrheagenic Escherichia coli* (DEC) (5.5%), rotavirus (3.9%), non-typhoidal *Salmonella* (NTS) (2.9%), and *Shigella* spp. (2.5%). The prevalence of *Shigella* spp. among rural patients (6.9%) was higher than that among urban patients (1.6%) (p < 0.001), with opposite trend for DEC (3.6% versus 5.9%, p = 0.007). An obvious seasonal pattern was observed for major pathogens, with peak for norovirus in autumn, rotavirus in winter and DEC, NTS, and *Shigella* spp. in summer. A wide variety of enteropathogens were detected among the elderly with acute diarrhea in China, with norovirus and DEC being the most commonly isolated pathogens. A strong seasonal pattern was observed for major pathogens of acute diarrhea among the elderly.

## Introduction

Diarrhea is one of the leading causes of morbidity and mortality worldwide. Although mortality rate of diarrhea among adults is lower than that in children less than 5 years [1], the burden of diarrhea in the elderly remains much high in a number of high-income countries, with the diarrhea-related deaths five times more than that in children in some circumstance [2]. In the United States, there was approximately 179 million patients of acute diarrhea each year, 83% of which deaths were the elderly [3]. In China, about 836 million patients of diarrhea occurred annually, with a population incidence rate of 56% [4]. To date, the etiology of diarrhea among Chinese children has been carefully described by a number of previous studies [4–7], while the characteristics of pathogens among the elderly in China with diarrhea remain poorly understood.

Last decade, the population of China has been aging considerably with a proportion of 10.1% (137.6 million) for those aged over 65 years in 2014 [8]. And it is estimated that the elderly will account for 25.3% in 2030 and 34.1% in 2050 in China [9]. Therefore, investigating the etiology of diarrhea among the elderly in China is an important public health priority for providing the evidence for disease prevention and control, vaccine development and policy recommendation in future. In this nationwide laboratory-based observational study, we detected pathogens, both bacterial and viral infections, for the acute diarrheal illness among patients aged over 65 years who presented to the hospital outpatient setting during 2009–2014.

## Materials and methods

### Ethics statement

This study was approved by the ethics review committee of National Institute for Communicable Disease Control and Prevention, Chinese Center for Disease Control and Prevention. The verbal consent was obtained from the patients, and the information on individual patients was strictly anonymized during the period of data analysis.

### Patient recruitment

From January 1, 2009 to December 31, 2014, a study of diarrhea for all age groups was conducted in 213 hospitals throughout mainland China. Study hospitals were selected based on their catchment areas, population served, and interest in participating in the study [5]. An episode of acute diarrhea was defined as ≥3 instances of watery, loose, mucus-containing or bloody stools within a 24-h period. A total sample size of approximately 10,000 persons per year was selected for the whole country with a median of approximately 54 patients per year by hospital. The study hospitals used their choice of convenient sampling methods to recruit participants among eligible patients. To account for seasonal variation in the incidence of diarrheal illness, each month per hospital sampled ≥5% of the allocated annual number. After obtaining verbal consent, demographic information (e.g., sex, age, and geographic location) and clinical characteristics (e.g., symptoms, date of symptom onset, and date of diagnosis) were collected using a standardized patient reporting form at the time of recruitment. In this study, the elderly was defined as the outpatients aged ≥65 years old. All patients who met our definition of elderly and recruited between 2009 and 2014 were included in the final analysis.

### Specimen collection

Two aliquots (one sample divided into two) of fecal specimens were collected from each patient by a trained nurse at each of the study hospitals. For virological testing, 5g of fresh whole stool was collected in sterilized containers without preservatives and stored at -20°C prior to processing. For bacteriological testing, fresh whole stool was collected using five sterilized cotton swabs and immediately placed in Cary -Blair Medium at 4°C for transportation. Collected specimens were packed and transported in ice boxes to laboratories in accordance with UN3373 transportation requirements within 24 h for bacterial tests, and within 48 h for viral tests.

### Enteropathogen testing

A total of 13 enteropathogens were included in this study: five viruses (adenovirus, astrovirus, norovirus, rotavirus and sapovirus) and eight bacteria [*Aeromonas hydrophila*, *Campylobacter* spp., diarrheagenic *Escherichia coli* (DEC), *Plesiomonas shigelloides*, non-typhoidal *Salmonella* spp. (NTS), *Shigella* spp., *Vibrio* spp., and *Yersinia* spp.]. A unified study protocol with standardized testing methods and operational procedures was performed in all study laboratories, which have been previously described in detail [5]. For rotavirus testing, fecal specimens were directly examined using enzyme-linked immunosorbent assay (ProSpecT^™^ Rotavirus kit, Oxoid Ltd, Basingstoke, UK) to confirm presence of Group A rotavirus antigens according to manufacturer’s instruction; RT-PCR was used for further genotyping of ELISA-positive samples [5]. For the remaining viruses, multiplex RT-PCR was used (Table 1). Bacterial testing employed two conventional culture approaches to isolate bacterial organisms from stool. The first was a direct isolation procedure; swabs from a Cary-Blair tube were plated directly onto Salmonellae-Shigella agar (SS), MacConkey (MAC) agar, xylose lysine desoxycholate (XLD) agar, and Campylobacter-selective agar plates (Karmali selective medium and Skirrow blood agar). Campylobacter-selective plates were incubated at 42°C under microaerophilic conditions; the remaining agar plates were incubated aerobically at 37°C. The second approach used enrichment procedures. Swabs were inoculated in selenite brilliant green sulfa enrichment broth (SBG), alkaline peptone water (AWP), m E. coli broth (mEC) and phosphate buffered saline (PBS). For Yersinia proliferation, inoculated PBS was incubated at 4°C for 10 days; all other enrichment broths were incubated aerobically at 37°C. Suspicious colonies formed on selective agar plates were further identified and characterized by morphological, biochemical, serological and genetic assays following standardized laboratory procedures.

**Table 1:** Primers and sequence information used in multiplex PCR for viral agents.

Virus	Primer	Polarity	Sequence (5’–3’)	Product Size (bp)	Reference
Rotavirus B	B5-2	+	GGCAATAAAATGGCTTCATTGC	814	[10]
	B3-3	−	GGGTTTTTACAGCTTCGGCT		
Rotavirus C	NG8S1	+	ATTATGCTCAGACTATCGCCAC	352	[11]
	NG8A2	−	GTTTCTGTACTAGCTGGTGAAC		
Adenovirus	Ad1	+	TTCCCCATGGCICAYAACAC	482	[11]
	Ad2	−	CCCTGGTAKCCRATRTTGTA		
Astrovirus	Mon269	+	CAACTCAGGAAACAGGGTGT	449	[12]
	Mon270	−	TCAGATGCATTGTCATTGGT		
Norovirus (GI)	G1-SKF	+	CTGCCCGAATTYGTAAATGA	330	[13]
	G2-SKR	−	CCRCCNGCATRHCCRTTRTACAT		
Norovirus (GII)	CoG2F	+	CARGARBCNATGTTYAGRTGGATGAG	387	[13]
	G2-SKR	−	CCRCCNGCATRHCCRTTRTACAT		
Sapovirus	SLV-5317	+	CTCGCCACCTACRAWGCBTGGTT	434	[13]
	SLV-5749	−	CGGRCYTCAAAVSTACCBCCCCA		

### Data analysis

The prevalence of a specific pathogen (defined as the proportion of patients that tested positive for each organism) was calculated by dividing the number of positive samples by the total number of samples that were tested for that pathogen. Patients were further divided into urban and rural groups based on their residential address according to the standards of the National Bureau of Statistics of China [14]. The seasons were defined as spring (March—May), summer (June—August), autumn (September—November), and winter (December—February), and the seasonality was assessed by fitting average monthly positive percentages to a linear regression model containing harmonic terms [15]. Binary logistic regression was used to compare the prevalence of enteropathogens between urban and rural patients. SPSS version 16.0 (SPSS Inc., Chicago, IL, USA) was used for data analyses. Chi-Square Tests was used to compare the proportions of different seasons. Results with a *p*-value < 0.05 were considered as statistically significant.

## Results

During the study period from January 1, 2009 to December 31, 2014, a total of 7,725 elderly outpatients with acute diarrhea were included in the study. The median age was 73 years [Inter Quartile Range (IQR): 68–79]. 4,106 (53.2%) were male. The median duration of diarrhea before visiting the doctor was 1 day (IQR: 1–2) and the median frequency of diarrhea was five times per day (IQR: 3–6).

At least one enteropathogen was detected from 1,617 (20.9%) of the 7,725 patients. The prevalence of enteropathogens is shown in Table 2. For single infections, the most commonly detected pathogens were norovirus (9.0%), followed by DEC (5.5%), rotavirus (3.9%), NTS (2.9%), and *Shigella* spp. (2.5%). Mixed infections were detected in 127 (1.6%) patients, with 114 of them due to dual infections. The most common mixed infections were norovirus combined with other enteropathogens, accounting for 47.2% (*n* = 60) of the mixed infections.

**Table 2:** Enteropathogens in the elderly outpatients (≥65 years) with acute diarrhea in China, 2009–2014.

Enteropathogens	Number of patients tested	Number of positive patients (%)^a^	Positive percentage (%)
**Viruses**^b^			
Norovirus	3867	346	9.0
G I	3867	28 (8.2)	0.7
G II	3867	315 (91.8)	8.2
Rotavirus	3857	149	3.9
Rotavirus A	3856	130 (87.3)	3.4
Rotavirus B	3867	7 (4.7)	0.2
Rotavirus C	3867	12 (8.1)	0.3
Astrovirus	3867	37	1.0
Adenovirus	3867	30	0.8
Sapovirus	3867	29	0.8
**Bacteria**^b^			
DEC	6122	339	5.5
ETEC	6122	79 (29.0)	1.3
EAEC	6122	78 (28.7)	1.3
EPEC	6122	72 (26.5)	1.2
EIEC	6122	31 (11.4)	0.5
EHEC	6122	12 (4.4)	0.2
NTS	6728	198	2.9
*S*. Enteritidis	6728	31 (24.4)	0.5
*S*. Typhimurium	6728	22 (17.3)	0.3
*Others*	6728	74 (58.3)	2.2
*Shigella* spp.	6417	157	2.5
*Shigella flexneri*	6417	112 (71.3)	1.8
*Shigella sonnei*	6417	33 (21.0)	0.5
*Shigella boydii*	6417	9 (5.7)	0.1
*Shigella dysenteriae*	6417	3 (1.9)	0.1
*Vibrio* spp.	6538	87	1.3
*Aeromonas hydrophila*	6236	56	0.9
*Campylobacter* spp.	5967	31	0.5
*Plesiomonas shigelloides*	6040	16	0.3
*Yersinia* spp.	6359	15	0.2
**Mixed infections**^c^	7725	127	1.6
Dual infection	7725	114	1.5
Triple infection	7725	11	0.1
Quadruple infection	7725	2	0.0

^a^ Percentage of genotypes/serotypes in genotyped/serotyped samples;

^b^ Only refers to single infections;

^c^ Denominators are total number of patients tested for one of 13 enteropathogens.

DEC, *Diarrhea-genic Escherichia coli*; NTS, Non-typhoidal *Salmonella spp*.; ETEC, *Enterotoxigenic E*. *coli*; EPEC, *Enteropathogenic E*. *coli*; EHEC, *Enterohaemorrhagic E*. *coli*; EAEC, *Enteroaggregative E*. *coli*; EIEC, *Enteroinvasive E*. *coli*.

GII was the most prevalent genogroup of norovirus detected, accounting for 91.8% (*n* = 315) of genotyped samples (*n* = 343). Group A was the predominant subtype of rotavirus isolated, accounting for 87.3% of all subtypes. Among the 80 genotyped Group A rotavirus strains, G9P [8] was the most frequent genotype identified, accounting for 20% (*n* = 16) of all isolates subtyped. Of the detected 272 DEC strains, 79 (29.0%) were enterotoxigenic *E*. *coli*, followed by enteroaggregative *E*. *coli* (*n* = 78, 28.7%), enteropathogenic *E*. *coli* (*n* = 72, 26.5%), enteroinvasive *E*. *coli* (*n* = 31, 11.4%), and enterohemorrhagic *E*. *coli* (*n* = 12, 4.4%), respectively. Of the 157 *Shigella* spp. isolates serotyped, the most frequently identified species was *S*. *flexneri* (*n* = 112, 71.3%) and *S*. *sonnei* (*n* = 33, 21.0%). Among the 127 serotyped NTS, *Salmonella* Enteritidis and *Salmonella* Typhimurium were the most commonly ones, accounting for 24.4% (*n* = 31) and 17.3% (*n* = 22), respectively (Table 2).

**Fig 1:**
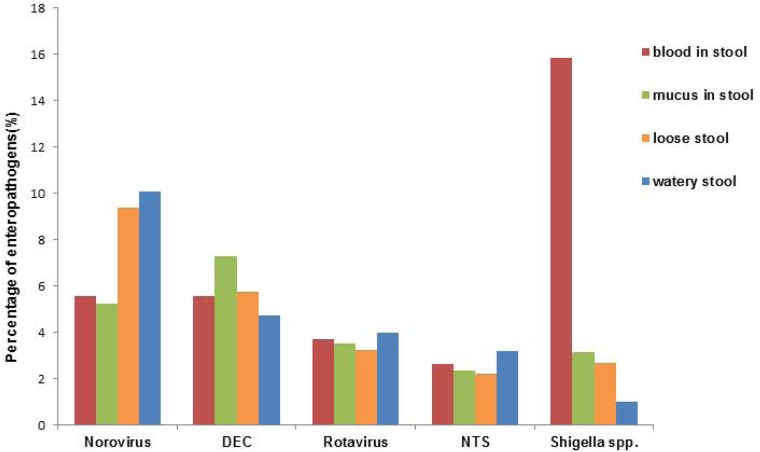
Positive proportion of predominant enteropathogens by stool type, 2009–2014. DEC = Diarrhea-genic Escherichia coli; NTS = Non-typhoidal *Salmonella* spp.

Among the 7,725 stool specimens collected from outpatients, watery stool (*n* = 3,960, 51.3%) was the most common type, followed by loose stool (*n* = 1,828, 23.7%), mucus stool (*n* = 1,321, 17.1%) and bloody stool (*n* = 296, 3.8%). Among these four groups of stool specimens, *Shigella* spp. (15.9%) was the most frequently detected pathogen from bloody stools (χ^2^ = 240.4, p < 0.001), while norovirus was more commonly associated with watery stool (10.1%) and loose stool (9.4%) (χ^2^ = 13.74, p = 0.003) (Fig 1).

Binary logistic regression analysis showed that the prevalence of both *Shigella* spp. and adenovirus in patients from rural areas was higher than that of those living in urban areas (*Shigella* spp.: OR = 4.68, 95% CI = 3.40–6.45, p < 0.001; adenovirus: OR = 3.07, 95% CI = 1.26–7.57, p = 0.015). Conversely, the prevalence of DEC in urban areas was higher than that of rural areas (OR = 1.66, 95% CI = 1.15–2.39, p = 0.007) (Table 3). No significant differences were found between urban and rural patients for the other 10 enteropathogens.

**Table 3:** Positive proportion of enteropathogens detected in urban and rural patients in China, 2009–2014.

Enteropathogens^a^	Positive percentage (%)		OR (95% CI)^b^	P value
	urban	rural		
**Viruses**				
Rotavirus	4.0	2.8	0.69 (0.33–1.41)	
Norovirus	8.9	9.2	1.03 (0.68–1.55)	
Sapovirus	0.8	0.3	0.43 (0.06–3.18)	
Adenovirus	0.7	2.0	3.07 (1.25–7.57)	0.015 (rural>urban)
Astrovirus	0.9	1.7	1.91 (0.74–4.93)	
**Bacteria**				
NTS	3.0	2.9	0.98 (0.67–1.44)	
*Shigella* spp.	1.6	6.9	4.68 (3.40–6.45)	<0.001 (rural>urban)
DEC	5.9	3.6	1.66 (1.15–2.39)	0.007 (urban>rural)
*Vibrio* spp.	1.2	1.8	1.47 (0.89–2.43)	
*Aeromonas hydrophila*	1.0	0.4	0.41 (0.15–1.13)	
*Campylobacter* spp.	0.6	0.2	0.35 (0.08–1.46)	
*Plesiomonas shigelloides*	0.3	-	-	
*Yersinia* spp.	0.2	0.5	2.44 (0.83–7.15)	

^a^ Single infection;

^b^ Rural patients were taken as the reference category for DEC, whereas urban patients were taken as the reference category for all other enteropathogens.

CI = confidence interval; NTS = Non-typhoidal *Salmonella* spp.; DEC = *Diarrhea-genic Escherichia coli*.

**Fig 2:**
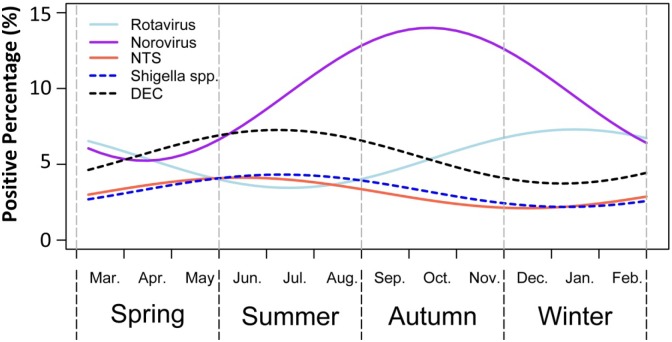
Seasonal pattern of predominant enteropathogens in the elderly with acute diarrhea in China, 2009–2014. DEC = Diarrhea-genic Escherichia coli; NTS = Non-typhoidal *Salmonella* spp.

The prevalence of norovirus, DEC, rotavirus, NTS, and *Shigella* spp. showed obvious seasonal patterns and which are significantly different among four seasons (p < 0.01). The prevalence of norovirus had a peak in autumn, while rotavirus had a peak in winter. In contrast, three bacterial agents, DEC, NTS, and *Shigella* spp., had a peak in summer (Fig 2).

## Discussion

This study has collected and analyzed the most comprehensive data to date on the etiology of diarrhea in the elderly across China. In this study, 20.9% of the recruited elderly outpatients with acute diarrhea were positive for at least one of 13 enteropathogens. Norovirus was the most commonly detected pathogen, followed by DEC, rotavirus, NTS, and *Shigella* spp. Comparing with patients living in urban areas, *Shigella* spp. had a higher prevalence in patients living in rural areas, while DEC had a lower prevalence in rural areas. The seasonal prevalence pattern varied among the detected pathogens, with peaks of norovirus in autumn, rotavirus in winter, and DEC, NTS, and *Shigella* spp. in summer.

Norovirus has been one of the most common pathogens associated with diarrhea in adults [16–18]. In this study, it was detected in 9.0% of patients, being the leading cause of acute infectious diarrhea in the elderly. 91.8% of norovirus strains were identified as norovirus GII, identical to many other studies [19–22] and demonstrating that GII strains are the most commonly isolated. DEC (5.5% of patients) was the second frequent pathogen detected in this study. This finding is comparable with those formerly reported in China [23–24], but which is much lower than South Africa (28.6%) [25] and the USA (9.4%) [26]. The prevalence of DEC would be affected by many factors including economic status, laboratory approaches, use of antibiotics prior to visiting hospital, specimen collection [5,27]. Further long-term detection is needed for better understanding the role of DEC in the elderly with actue diarrhea.

*Shigella* spp. had a higher prevalence in patients living in rural areas, while DEC had a higher prevalence in urban areas. This finding is similar to previous report in China [28]. Most researchers have reported that the etiology of viral diarrhea is similar between developed and developing countries [29–30]; however, we found that adenovirus had a higher prevalence in rural patients. These indicated the difference in etiology of diarrhea between developing and developed areas of China and require further study to explore. The seasonal pattern was also different between viruses and bacteria. Viruses were more prevalent in winter and autumn months, while bacteria were more prevalent in summer months. Seasonal pattern is likely associated with environmental factors [31–32], such as ambient temperature, precipitation, and humidity, all of which may influence exposure frequency, host immunity, and pathogenicity.

Our estimates of the prevalence of enteropathogens among elderly outpatients (20.9%) are much lower than previously estimated in outpatient children [5]. In total, 79.1% of samples did not yield any bacterial or viral pathogens. This high negative rate signifies the multi-factorial nature of acute diarrhea among the elderly. One possible reason was that some common gastrointestinal pathogens associated with diarrhea among the specific elderly population, such as *Clostridium difficile* [33–34], were not included in this study. The unidentified pathogens may account for an unknown fraction of the outpatients of the elderly with acute diarrhea in the present study, e.g. Spina et al. reported a positivity rate of 54% for 22 pathogens in Europe [35].

There were three main limitations in our study. Firstly, no all specimen submitted to laboratories were tested for all the 13 targeted pathogens, which probably have resulted in underestimate of positivity for certain enteropathogens. Nevertheless, this was the most comprehensive data collection on the etiology of diarrhea in the elderly in China to date. Secondly, the methods of virology test were different, ELISA for group A rotavirus and Multiplex PCR for the remaining viruses, which probably have resulted in slightly underestimate of positivity for group A rotavirus. Thirdly, only outpatients were recruited in this study, and inpatients who have a higher burden of disease were not analyzed. Therefore, we could not present the etiology of acute diarrhea among the hospitalized patients, and were uncertain about the clinical severity of diarrhea among the elderly population.

## Conclusions

In summary, in this 6-year prevalence study, we found that the most commonly detected enteropathogens in the elderly with acute diarrhea were norovirus and DEC. However, more causative agents of acute diarrhea among the elderly require further exploration. The findings demonstrated the complexity of the acute diarrhea among the elderly in China regarding of the features of pathogens, regional and seasonal diversity, etc. In the context of increasingly population aging in China, the research on disease burden and the high risk factors leading to hospitalization and death among the elderly due to acute diarrhea need to be further studied.
